# Developing the Message Assessment Scale for Tobacco Prevention Campaigns: Cross-sectional Validation Study

**DOI:** 10.2196/38156

**Published:** 2022-07-26

**Authors:** Jessica M Rath, Siobhan N Perks, Kenneshia N Williams, Tracy Budnik, John Geraci, Donna M Vallone, Elizabeth C Hair

**Affiliations:** 1 Truth Initiative Schroeder Institute Washington, DC United States; 2 Department of Health, Behavior and Society Johns Hopkins Bloomberg School of Public Health Baltimore, MD United States; 3 Department of Behavioral and Community of Health University of Maryland School of Public Health College Park, MD United States; 4 Bridge Analytics Limited Liability Company Mendham, NJ United States; 5 Crux Research Honeoye Falls, NY United States; 6 School of Global Public Health New York University New York, NY United States

**Keywords:** communication, youth/young adults, scales, message, behavior, health, campaign, tobacco, smoking cessation, prevention, youth, young adults, data, data analysis

## Abstract

**Background:**

Mass media campaigns are effective for influencing a broad range of health behaviors. Prior to launching a campaign, developers often conduct ad testing to help identify the strengths and weaknesses of the message executions among the campaign’s target audience. This process allows for changes to be made to ads, making them more relevant to or better received by the target audience before they are finalized. To assess the effectiveness of an ad’s message and execution, campaign ads are often rated using a single item or multiple items on a scale, and scores are calculated. Endorsement of a 6-item perceived message effectiveness (PME) scale, defined as the practice of using a target audience’s evaluative ratings to inform message selection, is one approach commonly used to select messages for antitobacco campaigns; however, the 6-item PME scale often does not produce enough specificity to make important decisions on ad optimization. In addition, the PME scale is typically used with adult populations for smoking cessation messages.

**Objective:**

This study includes the development of the Message Assessment Scale, a new tobacco prevention message testing scale for youth and young adults.

**Methods:**

Data were derived from numerous cross-sectional surveys designed to test the relevance and potential efficacy of antitobacco truth campaign ads. Participants aged 15-24 years (N=6108) responded to a set of 12 core attitudinal items, including relevance (both personal and cultural) as well as comprehension of the ad’s main message.

**Results:**

Analyses were completed in two phases. In phase I, mean scores were calculated for each of the 12 attitudinal items by ad type, with higher scores indicating more endorsement of the item. Next, all items were submitted to exploratory factor analysis. A four-factor model fit was revealed and verified with confirmatory factor analysis, resulting in the following constructs: personally relevant, culturally relevant, the strength of messaging, and negative attributes. In phase II, ads were categorized by performance (high/medium/low), and constructs identified in phase I were correlated with key campaign outcomes (ie, main fact agreement and likelihood to vape). Phase II confirmed that the four constructs identified in phase I were all significantly correlated with main fact agreement and vape intentions.

**Conclusions:**

Findings from this study advance the field by establishing an expanded set of validated items to comprehensively assess the potential effectiveness of advertising executions. This set of items expands the portfolio of ad testing measures for ads focused on tobacco use prevention. Findings can inform how best to optimize ad executions and message delivery for health behavior campaigns, particularly those focused on tobacco use prevention among youth and young adult populations.

## Introduction

Mass media public education campaigns are an effective population-level intervention for influencing a broad range of health behaviors. Prior to launching a campaign, developers often conduct message testing to assess creative executions that will be effective among the specific audience targeted by the campaign. For a campaign to be effective, messages must resonate with the target audience to influence changes in knowledge, attitudes, and intentions, and in turn, the specific health behavior of interest. According to the Centers for Disease Control and Prevention, when campaign messages are delivered to attain sufficient reach, frequency, and duration, one can expect changes in campaign-targeted attitudes after the campaign has aired for 12 to 18 months and can expect behavioral changes after the campaign has aired for 18 to 24 months [1]. To assess message effectiveness, campaign messages are often rated using a single item or multiple items on a scale, and scores are calculated [2].

Perceived message effectiveness (PME), defined as the practice of using a target audience’s evaluative ratings to inform message selection, is one approach to assess message effectiveness. Measuring PME using six items (ie, the ad is powerful, the ad is meaningful, the ad captures my attention, the ad is informative, the ad is convincing, and the ad is worth remembering) on a 5-point agreement scale has been shown to be a valid predictor of ad effectiveness [3]. The assumption is that messages scoring higher on the PME scale would be more likely to affect actual message effectiveness, for example, changing knowledge, attitudes, intentions, and ultimately the behavior of interest [4].

Several studies demonstrate the predictive validity of PME for changes in attitudes, intentions, and behaviors related to substance use among young people, particularly related to antitobacco campaigns [3,5-8]. For example, a recent systematic review of longitudinal studies in the antitobacco campaign literature examined the use of PME as a validated indicator of message effectiveness; researchers found that across 6 studies, PME provided predictive validity in measuring the effectiveness of antitobacco-related messages. In particular, the review confirmed that PME was associated with a variety of beliefs (eg, beliefs about smoking and quitting smoking) and behaviors (eg, message recall, conversations about ads, quit intentions, and cessation behavior) [8]. Although PME has been widely used for message selection, the scale often does not produce enough specificity to effectively modify or optimize an ad execution to improve its efficacy. Ad optimization refers to the process of using data to guide modifications/changes to ads that make them more relevant to or better received by the target audience. In addition to PME not providing enough specificity, PME has also primarily been used to assess smoking cessation media messages designed for adult populations.

The purpose of this study was to develop and validate additional measures to assess message effectiveness while providing additional evidence for ad optimization for tobacco prevention campaigns designed for youth and young adults. Data related to ad optimization, prior to campaign launch, can increase the likelihood of maximizing campaign resources. Findings from this study can broaden the capacity of evaluators to conduct message testing for antitobacco campaigns, particularly with respect to younger populations.

## Methods

### Overview

This study was conducted in two phases. Phase I used data from 11 cross-sectional surveys, conducted using the Dynata online panel from December 2016 to September 2018. Surveys were identical (with the exception of minor customizations, specific to the ads) and were conducted to assess receptivity to truth campaign ads that messaged on tobacco use but varied in their strategic goals. Participants were randomized to view only one ad, to avoid positional bias, before completing the survey. Each survey included 12 items related to message receptivity, including perceptions of effectiveness and relevance. The final sample included 2577 participants, aged 15-24 years, across 5 antitobacco ads (n=1275) and 6 antivape ads (n=1302). During this phase, mean scores were calculated for each of the 12 items by ad type, and factor analyses were run. Factor analyses were run at this phase to identify how the 12 items fit together and mapped onto different constructs.

The phase II analysis was conducted to validate constructs identified at phase I. Together, these constructs would go on to be referred to as the Message Assessment Scale. Phase II included the constructs identified at phase I in 13 cross-sectional forced exposure surveys, conducted using the Dynata online panel from July 2014 to June 2019 to test truth campaign ads. The final sample included 3531 participants, aged 15-24 years, across 10 antitobacco ads (n=2633) and 3 antivape ads (n=898). Mean scores for the constructs were examined across ads to assess campaign-aligned performance, and correlations were run to examine the relationship between construct scores for antivape ads, main fact agreement, and vape intentions. The goal of the correlation analysis at this phase was to determine if the constructs identified at phase I impacted campaign outcomes in a campaign-aligned direction.

### Ethical Considerations

All study protocols were reviewed and approved for human participation in research by the institutional review board of Advarra Inc (Pro00034056; formally Chesapeake IRB). Informed consent was included in the online survey and participants could check “Yes, I would like to continue” or “No, I do not wish to take part in this study.” Parental permission was collected from minor participants younger than 18 years.

### Measures

#### Attitudinal Items

Ad *likability* was assessed using the item “In general, what is your impression of this ad?” Response options were on a 5-point Likert scale including “dislike it a lot,” “dislike it somewhat,” “neither like nor dislike it,” “like it somewhat,” and “like it a lot.” Average likability was reported, ranging from 1 to 5, with higher scores indicating a more likable ad.

All other attitudinal items were assessed on a 5-point Likert scale including “strongly disagree,” “disagree,” “neither agree nor disagree,” “agree,” and “strongly agree.” The following base was used: “How strongly do you agree or disagree with each of the following statements about the ad?” Items included “It told me something I didn’t already know,” “It gave me good reasons not to [smoke; use e-cigarettes/vape],” “It really speaks to me,” “I identify with what this message says,” “It is for people like me,” “It is relevant for my generation,” “It feels modern/current,” “It is an acceptable way to talk about the issue of [smoking; using e-cigarettes/vaping],” “It is motivating,” “It is believable,” “It makes me want to tell someone about it,” “It is confusing,” “It is too fast,” and “It is offensive.”

#### Main Fact Agreement

Each of the 13 cross-sectional surveys used in phase II included main facts for the ad being tested. Participants were asked “How strongly do you agree or disagree that the video communicates each of the following messages?” Examples of facts included were “Just because vaping is safer than cigarettes, doesn’t make it safe” and “People who vape are being tested on.” Response options were on a 5-point Likert scale ranging from “strongly disagree that the ad conveys this message” to “strongly agree that the ad conveys this message.” Higher scores indicated greater fact agreement.

#### Vape Intentions

Self-reported change in likelihood to use or try vapes or e-cigarettes after seeing each ad was assessed by the item “Since having seen this ad are you...?” Answer options included “much more likely to use or try vapes or e-cigarettes including JUUL,” “somewhat more likely to use or try vapes or e-cigarettes including JUUL,” “no change in my likelihood to use or try vapes or e-cigarettes including JUUL,” “somewhat less likely to use or try vapes or e-cigarettes including JUUL,” and “much less likely to use or try vapes or e-cigarettes including JUUL.” Items were categorized for analysis into “more likely,” “no change,” and “less likely” to use or try vapes or e-cigarettes including JUUL.

#### Demographics

Demographic items included age (grouped as 15-17 years, 18-21 years, and 22-24 years), gender (male and female), race/ethnicity (non-Hispanic White; Hispanic, Latino, or Spanish origin; non-Hispanic Black or African American; and non-Hispanic other/declined), and subjective financial situation (do not meet basic expenses, just meet basic expenses with nothing left over, meet needs with a little left over, and live comfortably).

### Analysis Plan

Descriptive statistics were calculated to identify the phase I and phase II samples. In the phase I analysis, mean scores were calculated for each of the 12 attitudinal items by ad type, with higher scores indicating more endorsement of that item. The 12 attitudinal items were then entered into an exploratory factor analysis to determine possible factor structures. Factor loadings and subsequent interpretation of the relative component scores indicated that a four-factor solution best fit the data. Using the supported four-factor structure, a confirmatory factor analysis (CFA), using varimax rotation, was conducted to determine the validity of the scale, check model fit, and examine internal consistency for each identified factor using Cronbach alpha. Final factors and corresponding psychometric properties are presented to represent phase I results.

In phase II, an external marketing consultant, with a decade’s long experience analyzing truth pre- and postmarket data, placed the 13 ads into high (n=6 ads), medium (n=4 ads), or low (n=3 ads) performance groups based on the original goals of each advertisement such as agreement with key targeted beliefs like “ending youth smoking is an achievable goal” and relative performance of each against strategic objectives. Mean scores were examined across all ads in each ad performance group to determine how these four constructs varied by performance. Mean scores were also used to determine if items performed in a campaign-aligned direction such that more agreement on items indicated the ads were more relevant and message comprehension was higher. Finally, correlations were examined between construct scores for the antivape ads and main fact agreement as well as between construct scores for the antivape ads and self-reported change in likelihood (ie, a decrease in intentions) to vape since having seen the ad. Data were analyzed using SPSS (IBM Corp) and MPlus (Muthén and Muthén) statistical modeling programs.

## Results

### Sample Description

Participant characteristics are summarized in Table 1. At phase I, the mean age was 19.4 (SD 2.9) years, while at phase II, the mean age was 18.8 (SD 2.5) years. Of the 2577 participants in phase I, 1315 (51%) were male and 1262 (49.9%) were female. The majority (n=1378, 53.5%) were non-Hispanic White. Of the 3531 participants in phase II, 1793 (50.8%) were male and 1738 (49.2%) were female. Non-Hispanic White respondents also represented the largest racial category (n=1829, 51.8%). Finally, at phase I, most (n=1649, 64%) respondents reported meeting their financial needs either “comfortably” or with “a little left over,” and at phase II, most (n=2317, 65.6%) respondents also reported meeting their financial needs either “comfortably” or with “a little left over.”

**Table 1:** Sample characteristics for the phase I and phase II samples.

Characteristic			Phase I (n=2577), n (%)		Phase II (n=3531), n (%)
**Age (years)**					
	15-17	834 (32.4)		1193 (33.8)	
	18-21	1030 (40.0)		1833 (51.9)	
	22-24	713 (27.7)		505 (14.3)	
**Gender**					
	Male	1315 (51.0)		1793 (50.8)	
	Female	1262 (49.0)		1738 (49.2)	
**Race/ethnicity**					
	Non-Hispanic White	1378 (53.5)		1829 (51.8)	
	Hispanic, Latino, or Spanish origin	518 (15.3)		504 (14.3)	
	Non-Hispanic Black or African American	393 (20.1)		746 (21.1)	
	Non-Hispanic, Other/Declined	288 (11.2)		452 (12.8)	
**Subjective financial situation**					
	Do not meet basic expenses	191 (7.4)		248 (7.0)	
	Just meet basic expenses with nothing left over	737 (28.6)		966 (27.4)	
	Meet needs with a little left over	1071 (41.6)		1418 (40.2)	
	Live comfortably	578 (22.4)		899 (25.5)	

### Phase I

CFA results are summarized in Table 2. The factor analysis revealed four new constructs (personally relevant, culturally relevant, strength of message, and negative attributes) with a total of 12 items. Fit statistics indicated that the four-factor model fit the data well (comparative fit index 0.97, root mean square error of approximation 0.05, standardized root mean squared residual 0.03; χ^2^_66_=9182.5; *P*<.001) [9].

Mean scores for each of the 12 items tested in phase I are listed in Table 3 by ad type (antitobacco and antivape). Mean score analyses indicated that the items performed similarly across antitobacco and antivape ads. The personal relevance, cultural relevance, and strength of message items had higher overall mean scores (scores closer to 5 indicated more agreement that the ads were personally relevant, were culturally relevant, and had strong messaging). The negative attribute items, however, had lower overall mean scores, indicating less confusion, offensiveness, or pacing concerns.

**Table 2:** Confirmatory factor analysis results for the phase I sample.

		Estimate	SE	Est/SE
**Personally relevant**				
	It really speaks to me	0.83	0.01	83.50
	I identify with what this message says	0.73	0.01	55.50
	It is for people like me	0.72	0.02	49.24
**Culturally relevant**				
	It is relevant for my generation	0.75	0.02	48.31
	It feels modern/current	0.72	0.02	45.76
	It is an acceptable way to talk about the issue	0.75	0.02	50.76
**Strength of message**				
	It is motivating	0.81	0.01	74.20
	It is believable	0.73	0.01	51.19
	It makes me want to tell someone about it	0.74	0.01	56.79
**Negative attributes**				
	It is confusing	0.81	0.02	39.06
	It is offensive	0.57	0.03	22.97
	The pace was too fast	0.56	0.02	25.23

**Table 3:** Mean scores for the items tested in phase I, by ad type.

			Antitobacco ads (n=1275), mean (SD)		Antivape ads (n=1302), mean (SD)
**Personally relevant (range 1-5)**					
	It really speaks to me	3.36 (1.12)		2.99 (1.17)	
	I identify with what this message says	3.41 (1.12)		3.28 (1.09)	
	It is for people like me	3.64 (1.08)		3.17 (1.16)	
**Culturally relevant (range 1-5)**					
	It is relevant for my generation	3.97 (0.91)		3.78 (1.08)	
	It feels modern/current	3.92 (0.90)		3.55 (1.07)	
	It is an acceptable way to talk about the issue	3.93 (0.94)		3.65 (1.06)	
**Strength of message (range 1-5)**					
	It is motivating	3.69 (0.99)		3.25 (1.11)	
	It is believable	4.02 (0.88)		3.69 (1.01)	
	It makes me want to tell someone about it	3.49 (1.12)		3.21 (1.13)	
**Negative attributes (range 1-5)**					
	It is confusing	2.02 (1.05)		2.23 (1.07)	
	It is offensive	2.04 (1.09)		2.01 (0.99)	
	It is too fast	2.34 (1.02)		2.54 (1.06)	

### Phase II

Internal consistency for the constructs identified at phase I was assessed using Cronbach alpha. The four new constructs had alpha scores above .69, indicating an acceptable level of internal consistency (Table 4). Mean scores for the personal relevance, cultural relevance, and strength of message constructs were the highest for the high-performance ads. Accordingly, the mean scores for the negative attributes constructs were the lowest for the high-performance ads (Table 4).

Correlations with main fact agreement and vape intentions for the antivape ads are summarized in Tables 5 and 6. Overall, results revealed that the personal relevance, cultural relevance, and strength of message constructs were significantly positively correlated with main fact agreement and vape intentions for most of the antivape ads. In other words, as personal relevance, cultural relevance, and strength of message scores increased, participants were more likely to agree with the ads’ main fact and report a decrease in intentions to vape. Results also revealed that higher scores on the negative attributes construct showed an overall significant inverse relationship with main fact agreement and vape intentions.

**Table 4:** Mean scores for the four constructs tested in phase II, by ad performance level.

	Alpha	High performance (n=6 ads), mean (SD)	Medium performance (n=4 ads), mean (SD)	Low performance (n=3 ads), mean (SD)
Personally relevant^a^	.77	3.57 (0.21)^b^	3.45 (0.07)	3.08 (0.19)
Culturally relevant^a^	.79	3.80 (0.15)^c^	3.89 (N/A^d^)^e^	3.56 (0.25)
Strength of message^a^	.69	3.69 (0.23)	3.49 (0.08)	3.34 (0.21)
Negative attributes^a^	.75	2.18 (0.10)	2.24 (0.13)	2.55 (0.19)

^a^Range of possible scores for constructs was 1-5.

^b^n=5 ads in the “high performance” group for this item.

^c^n=2 ads in the “high performance” group for this item.

^d^N/A: not applicable.

^e^n=1 ad in the “medium performance” group for this item.

**Table 5:** Correlations with main fact agreement for the phase II antivape ads.

Ad tested	Antivape ad 1 (n=298), *r* (*P* value)	Antivape ad 2 (n=299), *r* (*P* value)	Antivape ad 3 (n=301), *r* (*P* value)
Personally relevant	0.20 (.001)	0.32 (<.001)	0.24 (<.001)
Culturally relevant	0.23 (<.001)	0.43 (<.001)	0.35 (<.001)
Strength of message	0.21 (<.001)	0.45 (<.001)	0.28 (<.001)
Negative attributes	–0.19 (.001)	–0.22 (<.001)	–0.23 (<.001)

**Table 6:** Correlations with vape intentions for the phase II antivape ads.

Ad tested	Antivape ad 1 (n=298), *r* (*P* value)	Antivape ad 2 (n=299), *r* (*P* value)	Antivape ad 3 (n=301), *r* (*P* value)
Personally relevant	0.21 (.004)	0.20 (.008)	0.13 (.11)
Culturally relevant	0.28 (<.001)	0.30 (<.001)	0.19 (.02)
Strength of message	0.23 (.002)	0.32 (<.001)	0.12 (.14)
Negative attributes	–0.02 (.78)	–0.32 (<.001)	–0.13 (.10)

## Discussion

### Principal Findings

Study findings demonstrate the utility of an expanded set of ad testing items to aid in message selection and optimization (four constructs). The set of validated constructs, when used together, are referred to as the Message Assessment Scale. The constructs can be used to assess each individual item or by calculating construct scores. These constructs provide useful specific data to inform how best to increase an ad’s relevance and effectiveness, specific to a youth and young adult audience. For example, an ad may perform low on cultural relevance, in which case qualitative responses to the overall advertisement “likes” and “dislikes” would be coded. The initial low score on this scale would tip researchers off to the need to dive deeper and look for comments in the qualitative questions, which could inform the low score and provide insight on how to improve it. The ads in this study that best met their goals were more personally and culturally relevant, and had stronger messaging than the lower performing ads. This is especially important because the validated constructs were significantly correlated with intentions not to vape for antivape ads. The better an ad performs on these constructs, the more potential it has to decrease vape intentions among a youth and young adult audience. Ad optimization, based on construct results, demonstrates the utility of maximizing message effectiveness for health behavior campaigns.

In Table 3, we see higher mean scores on the constructs for antitobacco (combustible products) versus antivape ads. These findings were not surprising given how long antitobacco (cigarette) ads have been on air, rates of smoking at that time, and the consensus around their impact on health. Constructs were developed from antivape ads airing early in the vaping epidemic, a period of time in which we were trying to stop a behavior that youth were really enjoying. An antivape message at that time felt less relevant to our audience because there was no data to indicate health or other negative implications from vaping yet.

The Message Assessment Scale can help establish benchmarks for modifying aspects of the execution, the placement of the execution, and the frequency of airing the execution. Ad testing, thus, informs placement and frequency decisions. For example, ads scoring higher on the measures may receive more or less frequent rotation on television or digital platforms, an ad on television or digital platforms may be coupled with another ad that increases scores, or a digital ad may be selected to be elevated to a television spot. Additionally, qualitative items related to ad receptivity and disapproval can be coupled with data from quantitative items to comprehensively assess likeability, level of novel information, and whether the execution provides motivation. For example, asking “what did you like most about the ad” and “what did you dislike about the ad” with open-ended responses can provide meaningful data that may not have been received from quantitative items with predetermined answer options. Moreover, the measures also provide insight into the possible reasons for low testing scores, including issues related to pacing and ad characteristics. If an ad is seen as offensive or confusing, this may interfere with the ad’s ability to effectively deliver a message to the target audience.

### Limitations

Although this study has many strengths, there are some limitations. First, the study used panel data, which does not reflect a probabilistic sample. As such, responses may not be generalizable to a broader population. However, sample quotas were set to yield approximately equal proportions by gender and age group. Additionally, this work did not examine correlations to in-market performance, which would ultimately test the ability of the constructs to predict how an ad may perform in the real-world.

### Conclusions

Findings advance the field by establishing an expanded set of validated items to comprehensively assess the potential effectiveness of advertising executions. The constructs can provide critical information for message optimization and message selection, particularly among a youth and young adult audience. Ensuring ads meet testing benchmarks before airing them across media platforms helps ensure cost efficiency while providing critical empirical evidence that messages will effectively shift knowledge, attitudes, and beliefs among the target audience. Future studies should explore whether the constructs perform similarly across demographic subgroups and correlate to actual campaign performance.

## Abbreviations

CFA: confirmatory factor analysis
PME: perceived message effectiveness
